# The Ionic and Metabolic Response Mechanisms of *Kochia scoparia* in Response to Saline–Alkaline Stress

**DOI:** 10.3390/plants14223540

**Published:** 2025-11-20

**Authors:** Xueyan Lu, Hui Fang, Qi Chen, Ying Zhang

**Affiliations:** 1 Heilongjiang Green Food Science Research Institute, Northeast Agricultural University, Harbin 150030, China; nefu20064764@126.com; 2 School of Resources and Environment, Northeast Agricultural University, Harbin 150030, China; zhangying_neau@163.com; 3 School of Life Sciences, Nantong University, Nantong 226010, China; fanghui8912@ntu.edu.cn

**Keywords:** *K. scoparia*, saline-alkaline stress, ionomics, metabolomics, phenolic compounds, mineral nutrients

## Abstract

Saline–alkaline stress is a critical environmental issue that limits plant growth and crop production. With the expansion of salinized land, investigating the response mechanisms of plants to salt–alkali stress is crucial. Integrated ionomic and metabolomic analyses were employed to investigate the response mechanisms of *Kochia scoparia* in our studies. Compared with the halophyte *Suaeda salsa*, *K. scoparia* exhibits distinct ionic and metabolic strategies for coping with saline–alkaline stress. Ca, Mg, and B were significantly accumulated in *K. scoparia* to alleviate ion toxicity and oxidative damage and to maintain cellular stability at the ionic element level. Sugars, alcohols, esters, and phenolic compounds were found to play key roles in resisting saline–alkaline stress at the metabolic level. Among these, sugars, alcohols, and esters were mainly involved in mitigating salt stress. Targeted metabolomic analysis indicated that certain phenolic compounds—namely C6C1-compounds (p-hydroxybenzoic, gallic, vanillic, salicylic, and syringic acids), C6C3 (caffeic acid, p-coumaric, p-hydroxycinnamic, cinnamic, and ferulic acids), and C6C3C6 (naringenin, quercetin, genistein, petunidin, and luteolin)—were significantly accumulated in *K. scoparia*. These compounds help mitigate saline–alkaline stress by enhancing reactive oxygen species (ROS) scavenging, modulating signaling pathways, reprogramming the osmoprotectant metabolism, and remodeling cell wall defense. This study elucidates the advantages and mechanistic of *K. scoparia*’s tolerance to saline–alkaline stress, providing a theoretical foundation for the repair and utilization of saline–alkaline soils.

## 1. Introduction

Saline–alkaline stress is a severe environmental issue that adversely affects plant growth, development, and productivity, thereby threatening the sustainability of land use [1,2,3]. Nearly 20–30% of the 1.5 billion hectares of cultivated land worldwide suffer from some degree of salinization [4]. In China, there are approximately 100 million hectares of salinized soil, and this area continues to expand on account of improper irrigation practices, climate change, and excessive fertilizer use [5]. Soil salinization poses a serious threat to land use and food security. Therefore, the effective improvement, development, and utilization of salinization soil are crucial pathways to ensuring food security and sustainable agricultural development [5,6].

A high salt concentration and elevated pH severely disrupt plant growth, causing ion toxicity, osmotic stress, oxidative damage, and nutrient imbalance [7,8,9]. These stresses decrease photosynthetic rates, arrest growth, reduce crop yield, and even cause plant death [10]. Plants surviving in highly saline–alkaline stress typically exhibit specialized ion accumulation characteristics [11]. Halophytes protect their growth from stress through specialized ion accumulation or exclusion mechanisms. Euhalophytes such as *S. salsa* maintain efficient K^+^ retention and employ efficient vacuolar Na^+^ sequestration, whereas recretohalophytes including *Limonium sinense* (Girard) Kuntze excretes excess ions externally [12,13]. Pseudohalophytes, such as *Phragmites australis*, restrict Na^+^ uptake at the root level [14]. This highlights distinct evolutionary strategies among halophyte functional groups. Despite these studies, the accumulation characteristics of ionic elements of different halophyte types remain poorly understood, limiting the identification of conserved ionic signatures and their application in engineering salt tolerance in crops.

Additionally, during prolonged exposure, plants produce complex metabolic responses that mobilize a large amount of metabolites—carbohydrates, amino acids, phenylpropanoids, flavonoids, and others—to survive the adverse environment [15,16,17,18,19,20,21,22]. Although these compounds are not required for basic development, they provide a rich reservoir of raw materials for pharmaceutical, agricultural, and industrial applications and play pivotal roles in plant stress adaptation [22,23,24,25,26,27]. In rice, for example, the over-expression of GROWTH-REGULATING FACTOR 7 (OsGRF7) increases arbutin content, and exogenous arbutin rescues the salt-sensitive phenotype of OsGRF7 knock-down and knock-out lines by suppressing salt-induced reactive oxygen species (ROS) accumulation [28]. Under salt stress, B specifically activates the phenylpropanoid pathway, enhancing the accumulation of antioxidants such as cinnamic acid, coumarin, and sinapic acid, together with flavonoids including glycitein and genistein, thereby collectively reducing ROS [29]. In soybeans, the over-expression of Gs5PTase8, an inositol polyphosphate 5-phosphatase cloned from wild soybean, lowers IP3 levels and improves salt tolerance in transgenic *Arabidopsis thaliana*, soybean hairy roots, and composite plants by limiting Na^+^ accumulation and maintaining a low Na^+^/K^+^ ratio [30].

*Kochia scoparia* (L.) Schrad. (*K. scoparia*), a typical salt-tolerant species, thrives widely in the saline–alkaline soils of northeastern China. Under saline–alkaline stress it exhibits strong tolerance to stress, making it a valuable model for dissecting the mechanisms that govern survival and defense allocation, knowledge that can be exploited to increase crop biomass on saline–alkaline land. However, the mineral elements and metabolic responses of *K. scoparia* to saline–alkaline stress remains poorly characterized, and the specific metabolites and ionic response mechanisms that underpin its stress resistance are still unknown.

In this study, we compared the responses of *K. scoparia* and *S. salsa* to saline–alkaline stress, using *S. salsa* as a reference. This work elucidates the adaptive mechanisms of *K. scoparia* to saline–alkaline stress, thereby facilitating crop improvement and the reclamation of saline lands. Key mineral elements and metabolites that are crucial for mitigating saline–alkaline damage were identified by integrating ionomics and metabolomics. Compared with *S. salsa*, *K. scoparia* employs a distinct tolerance strategy. At the ionic element level, *K. scoparia* preferentially accumulates Ca, Mg, and B to preserve cell stability and alleviate stress injury. At the metabolic level, elevated levels of sugars, alcohols, and esters were observed in *K. scoparia*, effectively counteracting salt stress. Further analyses indicated that phenolic compounds are central players in saline–alkaline resistance. These compounds activate the antioxidant machinery, modulate signaling cascades, reprogram the osmoprotectant metabolism, and reinforce cell wall defenses. These findings provide a novel breeding strategy for enhancing stress resistance and a practical route to an elite germplasm tailored for the restoration and productive use of saline soils.

## 2. Materials and Methods

### 2.1. Plant Materials and Sample Collection

**Figure 1:**
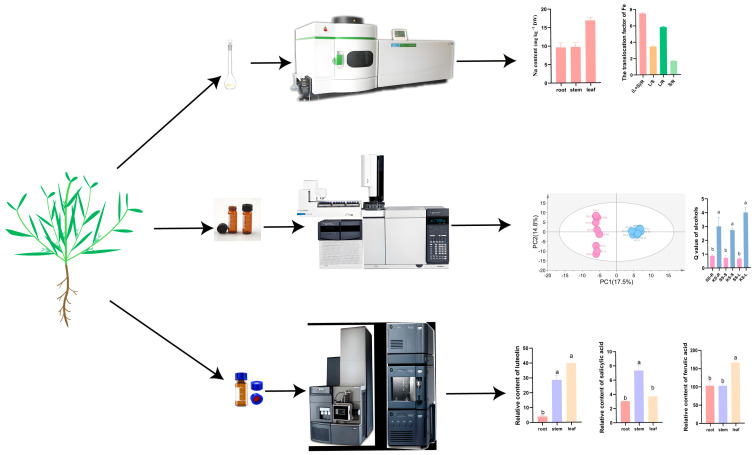
Experimental workflow diagram. Different letters represent significant differences.

*K. scoparia* was collected from the saline–alkaline grassland of Hulun Buir, Inner Mongolia Autonomous Region, China. In this study, all *Kochia scoparia* plants were collected from *S. salsa* communities. The habitats of *S. salsa* and *K. scoparia* are sulfate-dominated saline–sodic soils with pH > 9 (Table S1). Plants were sampled in July from three geographically distinct populations (>500 km apart), where *S. salsa* was the dominant co-occurring species. Five individuals were sampled from each *K. scoparia* population as one treatment, with each population serving as an independent replicate. The samples were frozen in liquid nitrogen and stored at −80 °C. The experimental workflow is summarized in Figure 1.

### 2.2. Element Detection in K. scoparia

To determine the element content of *K. scoparia*, the plants were divided into leaf, stem, and root. Three biological replicates were used for each part. The contents of B, Fe, Mn, Ni, Mo, Na, K, Ca, Mg, Cu, and Zn were determined following the method of Chen et al. [31]. The samples were dried at 60 °C for 48 h in a constant drying oven. The dried samples were pulverized. A 0.1 g portion of the powdered sample was soaked in 10 mL 95% HNO_3_. The mixture was heated on a graphite plate (EH45A plus, LabTech, Beijing, China) to achieve digestion at 130 °C and until the sample was completely clarified. The samples were adjusted to a final volume of 25 mL with the deionized water. The element content was detected using an inductively coupled plasma emission spectrometer (ICP-OES Optima 8000, Perkin Elmer, Waltham, MA, USA). The element content was calculated according to the standard curve. Factors of transfer factor (TF) were calculated by dividing the ionic element content of the above-ground tissues by the ionic element content of the root system [31].

### 2.3. Untargeted Metabolomics Detection in K. scoparia

Untargeted metabolomics analysis was performed as previously described [31]. Each group consisted of three biological replicates. In summary, 0.06 g of samples was homogenized with 400 μL of methanol (cold and 40 μL of internal standard solution). Subsequently, the samples were subjected to ultrasonication for 30 min and then mixed with 200 μL of chloroform and 400 μL of water. The mixture was centrifuged at 10,000× *g* for 10 min at 4 °C. Following centrifugation, 400 μL of the supernatant was collected and dried under vacuum at 20–25 °C. The residue was derivatized by adding 80 μL of methoxyamine, followed by incubation at 37 °C for 90 min. After adding 80 μL of BSTFA (including 1% TMCS) and 20 μL of n-hexane, the mixture was incubated at 70 °C for 60 min. The prepared sample was measured by the Agilent system (7890A-5975C GC-MS system, Agilent Technologies, Santa Clara, CA, USA). A non-polar DB-5 capillary column (30 m × 250 μm I.D., J&W Scientific, Folsom, CA, USA) was used for chromatographic separation, with high-purity helium as the carrier gas and a flow rate of 1.0 mL/min. The injection temperature was set to 260 °C and the ion source temperature was set to 230 °C. Electron impact ionization at −70 eV was conducted (full scan mode, *m*/*z* 30–600), with a data acquisition rate of 20 spectra/s. The QC sample was mixed samples from aliquots of various samples. Quality was controlled by internal standards. Potential false-positive peaks, known internal standards, and associated artifacts were excluded from the dataset. The dataset was normalized based on the total peak intensity within each sample.

### 2.4. Phenolic Compound Detection in K. scoparia

Phenolic compounds were determined using a Waters ACQUITY UPLC system (Waters, Milford, MA, USA) coupled to a quadrupole time-of-flight mass spectrometer (XEVO G2 QTOF, Waters), with the detailed protocol previously described in [32]. Next, 1.0 g samples were extracted via 10 mL 70% aqueous solution and homogenized, with ultrasound for 30 min. After centrifugation at 7200× *g* for 10 min at 4 °C, the retained supernatant was dried. Then, the sample was re-dissolved in 1 mL methanol (70%), with 0.22 μm nylon membrane filtration.

The UPLC-QTOF-MS operating conditions followed Chen et al. [32]. Each group consisted of three biological replicates. The ACQUITY UPLC BEH C18 column (1.7 mm, 2.1 mm, × 50 mm) was maintained at 30 °C and operated at a flow rate is 0.25 mL/min. The mobile phase consisted of A (0.1% formic acid–water) and B (0.05% formic acid–acetonitrile). MS conditions featured a scan range of atomizing air pressure, 25 psi, *m*/*z* 120–1200 with ESI source parameters including a spray voltage of 5500 V and a capillary temperature of 500 °C, and an air curtain pressure of 20 psi. The data were analyzed and normalized using MassLynxTM (Waters Corporation, Milford, MA, USA).

### 2.5. Statistical Analysis

The metabolites were subjected to multivariate statistical analyses (namely principal component analysis (PCA) and orthogonal partial least squares discriminant analysis (OPLS-DA)), to reveal changes in metabolic patterns among different groups. The variable importance in the projection (VIP) values were obtained from the OPLS-DA model. The metabolites with VIP values > 1 and *p*-values < 0.05 were considered as differentially accumulated metabolites. These differentially accumulated metabolites were annotated in the KEGG database to determine their functions and pathways of participation.

The principal component “Q” scores of different treatment groups were calculated using SPSS 24.0 statistical software (SPSS, Inc., Chicago, IL, USA). Hierarchical clustering analysis, histograms, heatmaps, and pathway maps were generated using the Wei Sheng Xin platform (https://www.bioinformatics.com.cn), GraphPad Prism8, and Microsoft Power Point. The data was presented as mean ± standard error (SEM).

## 3. Results

### 3.1. Element Accumulation and Translocation in K. scoparia

**Figure 2:**
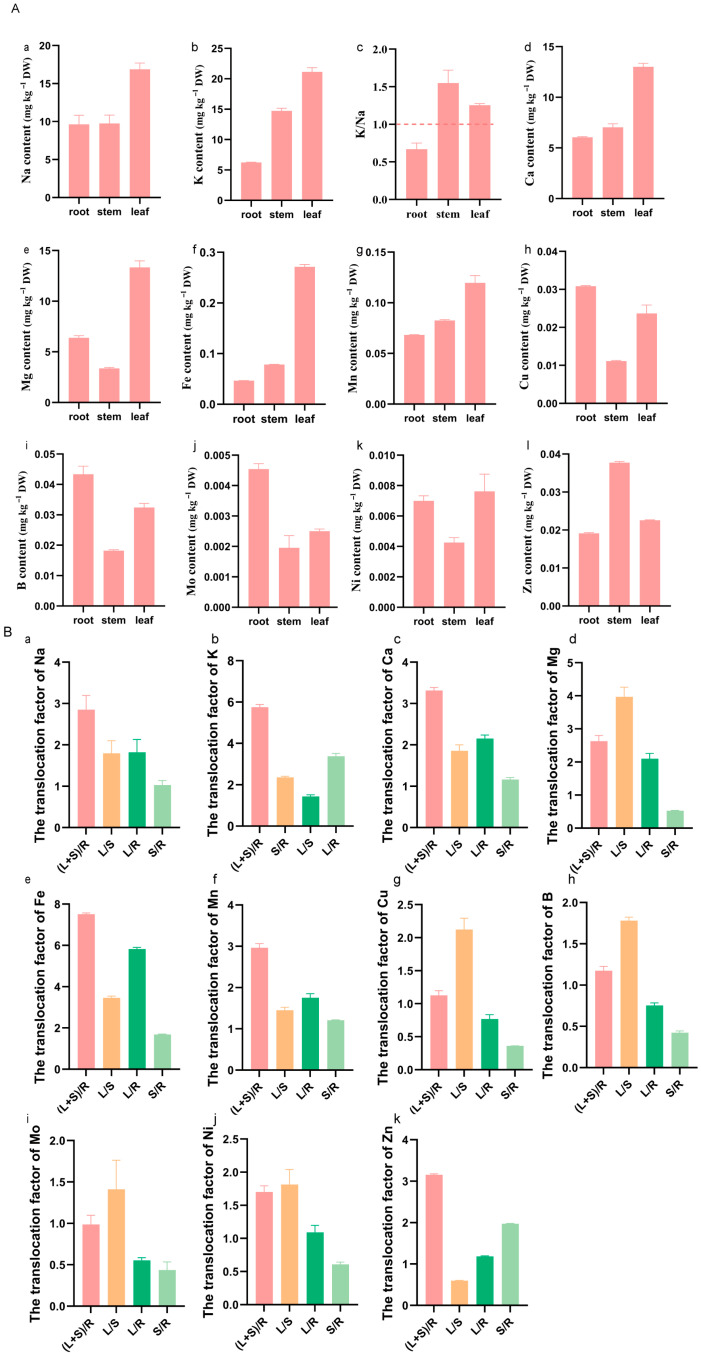
The accumulation and translocation factors of elements in *K. scoparia.* (**A**) The accumulation of elements Na (**a**), K (**b**), Ca (**d**), Mg (**e**), Fe (**f**), Mn (**g**), Cu (**h**), B (**i**), Mo (**j**), Ni (**k**), Zn (**l**) and K/Na ratio (**c**), (**B**) the translocation factors of elements Na (**a**), K (**b**), Ca (**c**), Mg (**d**), Fe (**e**), Mn (**f**), Cu (**g**), B (**h**), Mo (**i**), Ni (**j**), Zn (**k**). (L+S)/R, L/S, L/R, and S/R represent the translocation factors from roots to above-ground parts, stems to leaves, roots to leaves, and roots to stems, respectively.

Saline–alkaline stress can affect the accumulation and translocation of elements in plants. To examine this effect in *K. scoparia*, we measured Na, K, Ca, Mg, B, Fe, Mn, Ni, Mo, Cu, and Zn in different tissues. The K/Na ratio is commonly used to assess saline–alkaline tolerance. Our results showed that K and Na were preferentially allocated to the above-ground parts, where K consistently exceeded Na (Figure 2A(a–c)), yielding a K/Na ratio > 1 in both the leaves and stems and confirming the species’ inherent saline–alkaline tolerance (Figure 2A(a–c)). Similarly, Ca and Mg were clearly enriched in the above-ground parts, especially in the leaves (Figure 2A(d,e)), where their contents were three times those in the roots. Notably, Ca accumulation in *K. scoparia* exceeded that of the salt-tolerant *S. salsa* (Figure 2A(d), Table S2) [33]. Micronutrients displayed distinct patterns: Fe and Mn were significantly enriched in above-ground parts, particularly in the leaves (Fe reached 0.27 mg g^−1^ DW; Figure 2A(f,g)). Cu, B, Mo, and Ni were predominantly retained in the roots (Figure 2A(h–k)), with lower concentrations in the stems, implying that stems serve mainly as transport conduits. In contrast, Zn was markedly accumulated in the stems (Figure 2A(l)), a phenomenon also observed in *S. salsa* [33] and possibly related to the Zn-mediated alleviation of saline–alkaline stress.

Translocation factors (TFs) were calculated to quantify element-specific responses to saline–alkaline stress in *K. scoparia*. Nevertheless, TFs differed markedly among elements (Figure 2B). Root-to-shoot TFs for K and Fe reached 5.8 and 7.5, respectively (Figure 2B(b,c)), mirroring the high values reported for *S. salsa* [33]. In contrast, B and Mo TFs barely exceeded 1 (Figure 2B(h,i); Table S2), a pattern distinct from that observed in *S. salsa* [28]. Element allocation followed tissue-specific routes: Fe, K, Ca, and Mg were preferentially transferred to leaves, whereas Zn was directed mainly to stems (Figure 2B(b–e)). Cu, B, and Mo exhibited low transfer to either the leaves or stems (Figure 2B(h,i)). Except for Zn and Mo, all elements accumulated more in the leaves than in the stems. This differential distribution is presumably linked to growth demands and metal requirements under saline–alkaline stress. By reallocating elements among tissues, *K. scoparia* alleviate stress injury—a key component of its saline–alkaline tolerance strategy.

### 3.2. Metabolic Response of K. scoparia to Saline–Alkaline Stress

**Figure 3:**
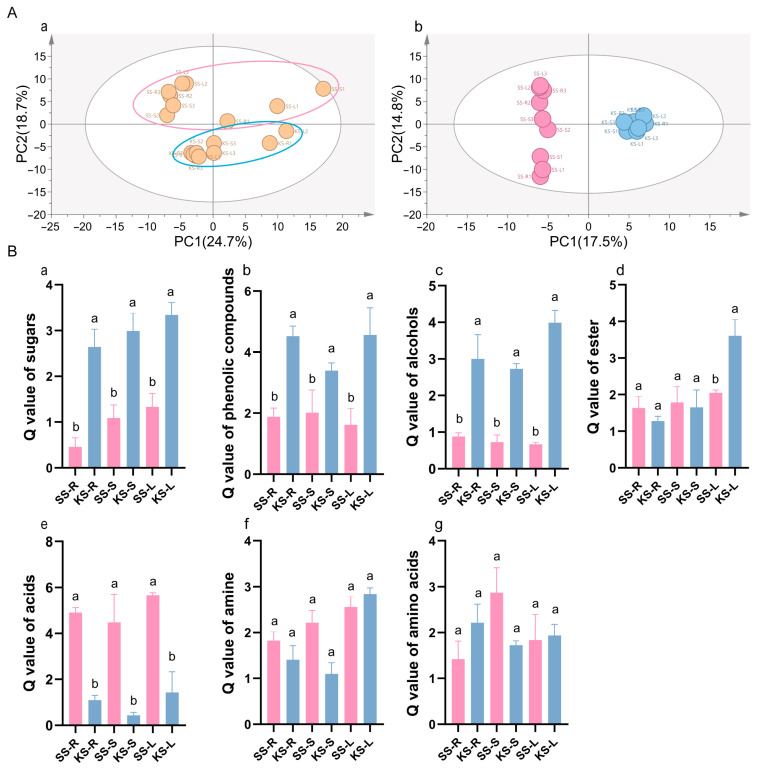
Metabolic divergence between *K. scoparia* and *S. salsa.* (**A**) PCA and OPLS-DA score plots of metabolite profiles. (**B**) Q-values for annotated metabolite classes. SS-R, SS-S and SS- denote roots, stems, and leaves of *S. salsa* (SS); KS-R, KS-S and KS-L denote roots, stems, and leaves of *K. scoparia* (KS). Data are means ± SE of three biological replicates; different letters represent significant differences (*p* < 0.05).

The accumulation of metabolites in the roots, stems, and leaves under different treatments was profiled using untargeted metabolomics. PCA and OPLS-DA score plots showed the tight clustering of biological replicates and clear separation among treatments (Figure 3A(a)); PC1 and PC2 explained 24.7% and 18.7% of the variance, respectively. A total of 204 metabolites were detected, of which 83 were significantly different (VIP > 1, *p* < 0.05) among groups (Figure 3A(b)). Chemical classification assigned these metabolites to seven major classes: amino acids (9), sugars (5), organic acids (28), alcohols (11), esters (7), amines (6), and phenolic compounds (6). The Q-values for sugars, phenolics, and alcohols were markedly higher in *K. scoparia* than in *S. salsa*, whereas organic acids were more abundant in *S. salsa* (Figure 3B(a–e)); the differences in amines and amino acids were not significant (Figure 3B(f,g)), indicating species-specific metabolic strategies.

**Figure 4:**
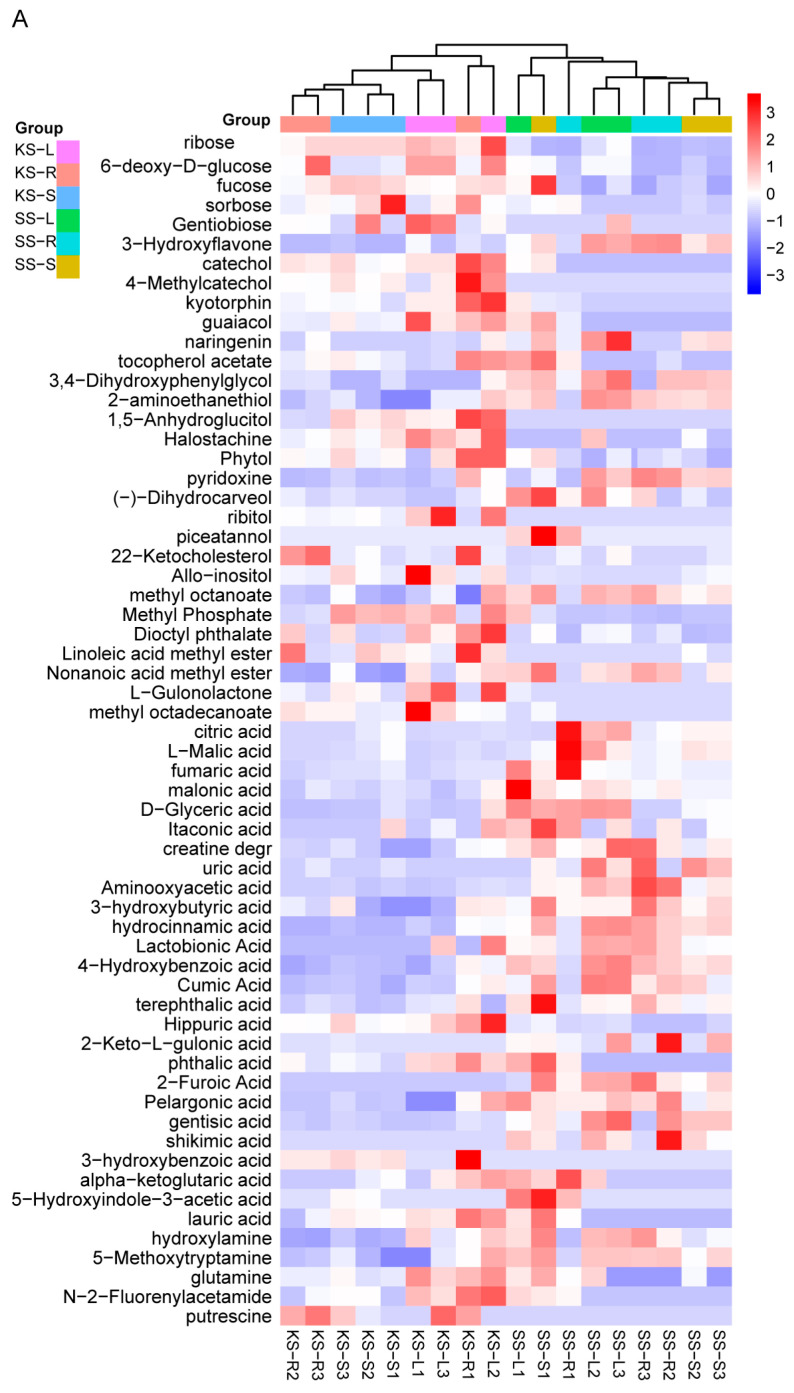

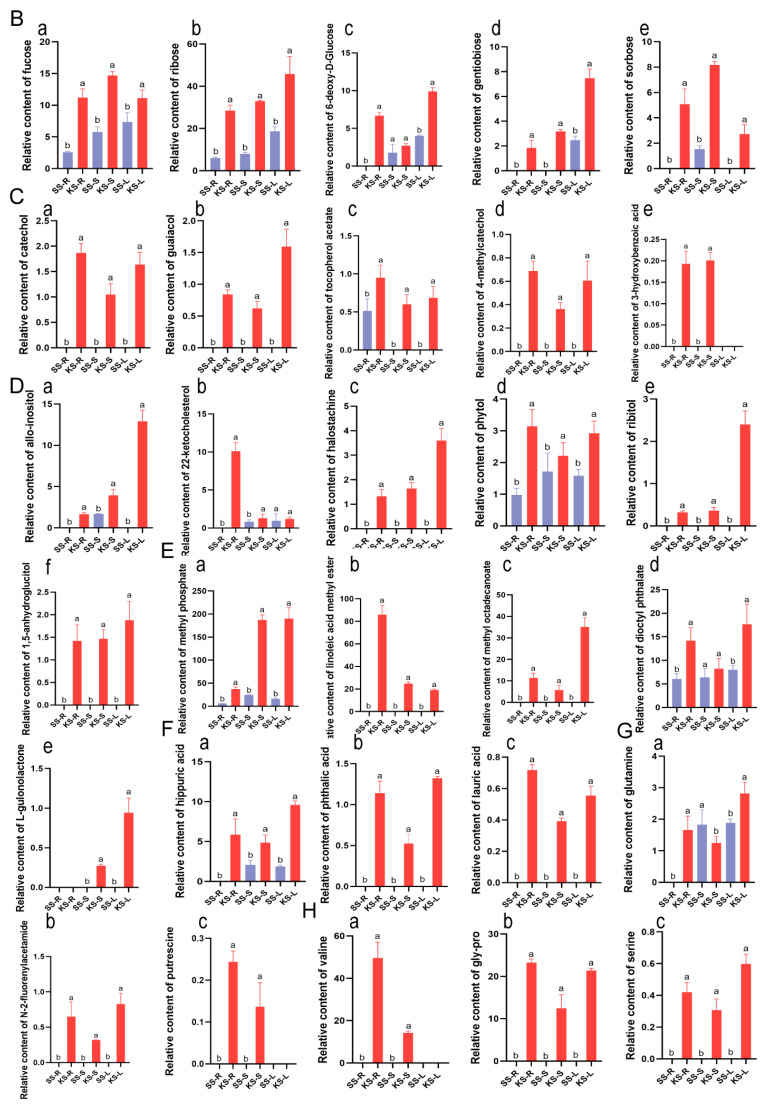
Differentially accumulated metabolites in *K. scoparia.* (**A**) Hierarchical clustering heatmap of significantly different metabolites across tissues. Relative contents in *K. scoparia* of (**B**) sugars, (**C**) phenolic compounds, (**D**) alcohols, (**E**) esters, (**F**) organic acids, (**G**) amines, (**H**) amino acids. SS-R, SS-S and SS-L denote roots, stems, and leaves of *S. salsa* (SS); KS-R, KS-S and KS-L denote roots, stems, and leaves of *K. scoparia* (KS). Significantly different metabolisms are summarized from three biological replicates and presented as the mean ± standard error. Different letters represent significant differences (*p* < 0.05).

Hierarchical clustering revealed tissue-wide accumulation patterns (Figure 4A). Relative sugar levels were consistently higher in all *K. scoparia* organs (Figure 4B). Among secondary metabolites, phenolic compounds were conspicuous: four of six (catechol, 4-methylcatechol, guaiacol, and tocopherol acetate) were significantly enriched in *K. scoparia* (Figure 4C). Likewise, six of eleven alcohols (1,5-anhydroglucitol, halostachine, phytol, ribitol, 22-ketocholesterol, and allo-inositol) and five of seven esters (methyl phosphate, dioctyl phthalate, linoleic acid methyl ester, L-gulonolactone, and methyl octadecanoate) accumulated preferentially in *K. scoparia* (Figure 4D,E).

By contrast, acids were enriched in *S. salsa*: 22 acid species accumulated preferentially in that species, whereas only 3—hippuric, phthalic, and 3-hydroxybenzoic acids—were more abundant in *K. scoparia* (Figure 4F). Overall, the Q-value of amines and amino acids did not differ significantly between the two species (Figure 3B). Nevertheless, gly-pro, serine, and valine were significantly higher in *K. scoparia* (Figure 4G), as were the amines putrescine, N-2-fluorenylacetamide, and glutamine (Figure 4H). Thus, *K. scoparia* metabolism is characterized by four dominant categories—sugars, phenolics, alcohols, and esters—all of which function in osmoregulation and general stress defense.

### 3.3. Phenolic Compounds Response of K. scoparia to Saline–Alkaline Stress

Motivated by the untargeted metabolomics results and the protective roles of phenolic compounds, we used LC-qTOF-MS to quantify these compounds across *K. scoparia* tissues. Of the 38 phenolic compounds targeted, 15 were reproducibly detected. L-phenylalanine—the universal precursor of the phenylpropanoid pathway—accumulated strongly, especially in above-ground organs (Figure S1). The remaining 14 compounds were grouped into three structural classes: C6C1-, C6C3-, and C6C3C6-compounds.

**Figure 5:**
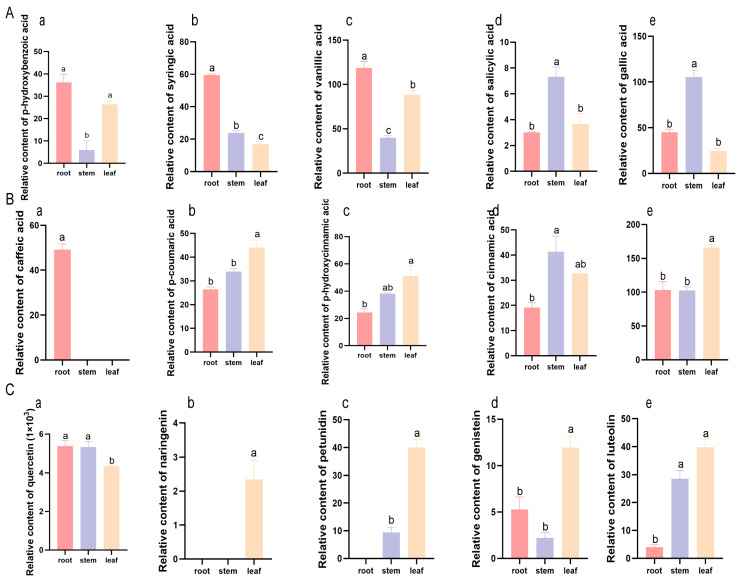
Relative content of phenolic compounds in *K. scoparia.* (**A**) Relative content of C6C1-compounds. Relative content of p-hydroxybenzoic acid (**a**), syringic acid (**b**), vanillic acid (**c**), salicylic acid (**d**) and gallic acid (**e**). (**B**) Relative content of C6C3-compounds. Relative content of caffeic acid (**a**), p-coumaric acid (**b**), p-hydroxycinnamic acid (**c**), cinnamic acid (**d**) and ferulic acid (**e**). (**C**) Relative content of C6C3C6-compounds. Relative content of quercetin (**a**), naringenin (**b**), petunidin (**c**), genistein (**d**) and luteolin (**e**). Different letters indicate significant differences among treatments (*p* < 0.05).

Five C6C1-compounds—p-hydroxybenzoic acid, syringic acid, vanillic acid, salicylic acid and gallic acid—accumulated preferentially in the roots and stems of *K. scoparia* (Figure 5A). P-hydroxybenzoic acid, syringic acid and vanillic acid, were most abundant in the roots, whereas gallic acid and salicylic acid peaked in the stems (Figure 5A). Among the C6C3-group, caffeic acid was root-specific; the remaining members (p-coumaric acid, p-hydroxycinnamic acid, cinnamic acid, and ferulic acid) were significantly enriched in leaves and stems (Figure 5B). C6C3C6-compounds accumulated in the above-ground parts of *K. scoparia*, except for quercetin, which was abundant in the stems and roots; naringenin and petunidin were restricted to the stems and leaves (Figure 5C). These phenylalanine-derived phenolic compounds thus accumulate tissue-specifically as part of the saline–alkaline response.

## 4. Discussion

Soil salinization is a major environmental threat to crop yield and ecological security [34]. The excess soluble salts in saline–alkaline soils inhibit plant growth, reduce productivity, and can be lethal [35,36]. Phytoremediation offers a promising route to reclaim such soils, yet the tolerance mechanisms of many halophytes remain unclear. Here we examined *K. scoparia* collected from the Hulunbuir Grassland, quantifying element and metabolite accumulation to elucidate its response to saline–alkaline stress.

### 4.1. The Response of Elements in K. scoparia to Saline–Alkaline Stress

*K. scoparia* exhibits robust vitality and is widely distributed across the severely saline–alkaline soils of the Hulunbuir Grassland. Compared with the halophyte *S. glauca*, it exhibits Na and K transport capacities close to those of *S. glauca*. (Figure 2, Table S2), although the total leaf accumulation of these elements remains lower than in *S. glauca*. Nevertheless, *K. scoparia* significantly outperforms *P. tenuiflora*, a species renowned for its saline–alkaline tolerance, in both K uptake and root-to-shoot translocation [31]. Moreover, the K and Na TFs in *K. scoparia* are similar to those in *S. glauca* (Figure 2, Table S2), indicating that *K. scoparia* can absorb and transport Na without disrupting K homeostasis—a key criterion for saline–alkaline tolerance. Although its K-uptake capacity is lower than that of *S. glauca*, it significantly exceeds that of *P. tenuiflora*. Thus, *K. scoparia* combines high K acquisition with efficient Na translocation, making it a valuable species for the phytoremediation of saline–alkaline soils.

Notably, our results show that Ca and Mg clearly accumulated in the above-ground parts of *K. scoparia*, especially in the leaves (Figure 2), even exceeding the levels in *S. salsa* (Figure 2, Table S2). This indicates that Ca and Mg play key roles in *K. scoparia*’s response to saline–alkaline stress. Ca functions as both an essential nutrient and a signaling hub that protects plants against saline–alkaline stress [37]. Through multidimensional and network-based regulation, it helps plants perceive stress, transmit signals, and execute resistance strategies, thereby enhancing salinity tolerance [38,39]. For example, exogenous Ca alleviates the oxidative stress caused by salt stress in peanut seedling roots by regulating the antioxidant enzyme system and flavonoid biosynthesis [39]. Different from Ca, Mg focuses more on sustaining the most fundamental and core physiological and metabolic processes. An adequate supply of Mg, serving as an activator for numerous enzymes, ensures the normal operation of essential metabolism under saline–alkaline stress [40,41]. Studies have shown that Mg-doped carbon dots can effectively remove ROS, enhance photosynthesis in rice, and protect it from oxidative damage caused by NaCl treatment [40].

Trace elements also contribute substantially to *K. scoparia*’s defense against saline–alkaline stress. Fe and Mn are efficiently translocated to the above-ground parts (Figure 2), where they serve as catalytic centers of antioxidant and photosynthetic enzymes that maintain cellular function under salt stress; comparable patterns have been reported in cotton, soybean, and rice [42,43,44]. In contrast to Fe and Mn, Cu must be tightly regulated, because both its deficiency and excess are phytotoxic. As a cofactor of superoxide-dismutase and plastocyanin, Cu is indispensable for scavenging ROS and sustaining electron transport, while it also promotes the lignification of root cell walls, forming a physical barrier that limits Na^+^ influx and water loss [45]. In our study, Cu was predominantly retained in *K. scoparia* roots, where it was used to reinforce cell wall lignification. This root-specific enrichment constitutes a key defensive strategy against saline–alkaline stress. For example, Cu oxide nanoparticles improved the physiological and biochemical response of *Arabidopsis thaliana* to salt stress, enhancing its salt tolerance [46].

Similarly to Cu, B and Mo were preferentially stored in *K. scoparia* roots (Figure 2). B maintains the mechanical strength of the cell wall, thereby reinforcing the physical barrier that preserves cellular integrity; its deficiency causes solute leakage and reduces osmoregulatory capacity [47,48,49]. Exogenous B has been shown to enhance root antioxidant activity and phenylpropanoid flux, decrease ROS, and retain Na^+^ in the roots, alleviating foliar oxidative damage and sustaining photosynthesis in salt-stressed soybean [24]. Mo—present in the lowest concentration of any trace element—is indispensable for nitrogen metabolism. It mitigates saline–alkaline stress-induced damage by ensuring efficient nitrogen metabolism, which supplies plants with sufficient energy and biosynthetic precursors to establish an effective antioxidant defense system [50,51]. In tomato, the salt-induced accumulation of Mo via SlMOT1 modulates ABA biosynthesis, stomatal conductance, and leaf Na^+^/K^+^ homeostasis [29]. Our results show that Mo accumulates mainly in *K. scoparia* roots, consistent with its known physiological role. For Ni, also linked to nitrogen metabolism, an adequate level mitigates N toxicity, enhances N assimilation, and improves ionic homeostasis [51]; in *Solanum lycopersicum* it alleviates salt stress by activating antioxidant enzymes and ion homeostasis [52]. Its above-ground enrichment in *K. scoparia* implies that Ni is actively recruited to sustain N-dependent processes under saline–alkaline conditions.

Interestingly, our results show that the trace element Zn accumulates specifically in *K. scoparia* stems, implying a distinctive role in saline–alkaline tolerance. Zn is an essential cofactor of numerous proteins and is required for auxin biosynthesis [51]; consequently, it acts as a growth promoter under saline–alkaline conditions [53]. Zn supplementation promoted salt tolerance in rice seedlings, giving treated plants a higher tiller height, dry weight, and fresh weight under salt stress [53,54]. In wheat, Zn treatment reduced oxidative stress and stimulated root, shoot, and spikelet growth, while increasing the levels of photosynthetic pigments, proline, total phenolics, and total carbohydrates relative to untreated controls [55].

**Figure 6:**
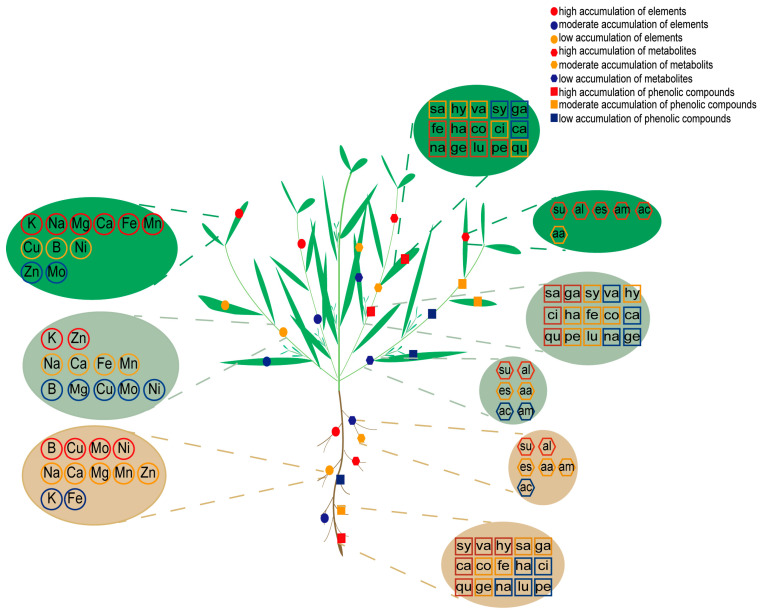
Elemental and metabolic responses of *K. scoparia* to saline–alkaline stress. The green background of the ellipse represents the accumulation in the leaves. The light green background of the ellipse represents the accumulation in the stems. The brown background of the ellipse represents the accumulation in the roots. The red circles, squares, and pentagons represent high accumulation. The orange circles, squares, and pentagons represent moderate accumulation. The blue circles, squares, and pentagons represent moderate accumulation.

In summary, our study reveals the elemental response signature of *K. scoparia* under saline–alkaline stress. Unlike *S. glauca*, *K. scoparia* allocates additional energy and resources to accumulating Ca, Mg, and key trace elements as part of its stress adaptation strategy (Figure 6).

### 4.2. The Response of Metabolites in K. scoparia to Saline–Alkaline Stress

The specificity of ion accumulation serves as a trigger for metabolic re-programming that enables plants to cope with saline–alkaline stress, while the subsequent reallocation of metabolites reinforces ionic homeostasis under these adverse conditions. To identify these key metabolites, we performed metabolomic profiling. Consistent with our earlier findings for *S. salsa*, acids accumulated extensively to buffer cytoplasmic pH [33]. In contrast to *S. glauca*, *K. scoparia* adopts a distinct metabolic strategy: sugars markedly accumulated in all tissues. These carbohydrates serve as both an energy supply and osmo-/signal molecules that modulate stress responses and act as osmore gulators to alleviate stress-induced damage [56]. Compared with *S. glauca*, most sugar-related metabolites accumulated to higher levels in *K. scoparia* (Figure 2), suggesting that sugar accumulation contributes to a differential response pattern to saline–alkaline stress between them. Sugar metabolism was shown to be widely linked to salinity tolerance in halophytes [56,57,58]. The significant accumulation of sugars in *K. scoparia* reflects an enhanced allocation of resources toward salt stress mitigation under saline–alkaline conditions.

Phenolic compounds were prominent among the secondary metabolites in *K. scoparia* (Figure 2). 3-hydroxyflavone, catechol, 4-methylcatechol, guaiacol, and tocopherol acetate were significantly elevated in our studies. These metabolites play multiple functions in plants coping with saline–alkaline stress, such as antioxidant defense, signal transduction, and osmotic adjustment [35,59]. The enrichment of these phenolic compounds emphasizes the importance of phenolic compounds for *K. scoparia*, although the well-defined mode of action remains to be explored. Alcohols are key osmotic regulators that help plants retain water under stress [33,60]; in our study, alcohols also accumulated significantly in *K. scoparia* compared with *S. salsa* (Figure 2). They can maintain cellular osmotic pressure, scavenge ROS, and protect biomolecules from oxidative damage [60,61].

Under saline–alkaline stress, esters perform various protective functions in plants, particularly in preserving membrane integrity, adjusting osmotic balance, and eliminating ROS [62]. For example, unsaturated fatty acid esters can enhance membrane fluidity and prevent membrane lipid peroxidation caused by saline–alkaline stress. Under salt stress, carbon flux was found to move toward triacylglycerol synthesis as an energy reserve to mitigate osmotic stress in plants [63,64]. There have been reports that the application of lactones can enhance the salt tolerance of wheat [65]; this is consistent with our experimental results. Esters play a functional role in the exposure of *K. scoparia* to salt stress.

The distinct metabolic adaptation strategies between *K. scoparia* and *S. salsa* were elucidating using metabolomics under saline–alkaline stress. Diverging from *S. salsa*’s alkaline stress-focused strategy, *K. scoparia* showed salt adaptation characterized by an increased content of osmolytes (sugars, alcohols, esters), with the concomitant up-regulation of phenolic compounds (Figure 2 and Figure 4).

### 4.3. The Response of Phenolic Compounds in K. scoparia to Saline–Alkaline Stress

Secondary metabolism plays a core role in the plant antioxidant system that contributes to stress tolerance [66,67]. Metabolomic profiling highlighted phenolic compounds as being especially abundant in *K. scoparia* (Figure 4). Phenolic compounds are crucial secondary metabolites for plants to respond to environmental stresses [68]; here they accumulated in large quantities, with L-phenylalanine as the dominant precursor (Figure 5 and Figure S1). L-Phenylalanine contributes to the synthesis of numerous defensive compounds and plays a key role in environmental adaptation to stresses such as saline–alkaline, drought, cold, and UV-B [69,70,71]. The abundant accumulation of L-phenylalanine in *K. scoparia* indicates that the phenylpropanoid biosynthesis pathway is critically involved in the adaptation to saline–alkaline stress.

Under saline–alkaline stress, C6C1-compounds—p-hydroxybenzoic, gallic, vanillic, salicylic, and syringic acids—accumulated significantly in *K. scoparia* roots and stems (Figure 5). Previous studies have demonstrated that plants subjected to periodic or recurrent saline–alkaline stress exhibit an enhanced accumulation of phenylpropanoid compounds, which subsequently strengthens their responsive role in long-term stress adaptation [29,72]. Salt-induced C6C1-biosynthesis reallocates carbon to ROS detoxification and tissue reinforcement [73], and these compounds function as both antioxidants and signaling molecules to alleviate saline–alkaline toxicity [74]. Exogenous p-hydroxybenzoic, salicylic, gallic, vanillic, and syringic acids have all been shown to mitigate salt-induced damage in tomato, rice, sunflower, and cucumber [75,76,77,78,79].

Ferulic acid, p-coumaric acid, p-hydroxycinnamic acid, cinnamic acid, and caffeic acid—classified as C6C3-compounds—accumulated mainly in the above-ground tissues of *K. scoparia* (Figure 5). These central phenylpropanoid pathway products combat saline–alkaline stress through antioxidant activity, ion homeostasis control, signaling, osmoregulation, cell wall modification, and microbial interactions [31,80,81]. External caffeic acid treatment significantly enhances the antioxidant enzyme system activity of wheat seedlings under salt stress [82]. Ferulic acid treatment reduces Na^+^ accumulation and enhances the K^+^/Na^+^ ratio in rice roots to resist salt stress [83].

In addition, C6-C3-compounds also help maintain cellular water balance by promoting the accumulation of osmoregulatory substances. For instance, p-coumaric acid increases soluble sugar content, reduces protein denaturation, and preserves enzyme activity under saline–alkaline stress [83]. Cinnamic acid effectively alleviates salt stress damage in peppermint by significantly enhancing antioxidant enzyme activities and osmolyte accumulation [84]. C6-C3-compounds also serve as precursors for lignin biosynthesis; their accumulation strengthens cell wall mechanical properties, thereby reducing water loss and ion leakage under saline–alkaline conditions [85]. Under salt stress, plants can reshape cell wall differentiation and modification via p-coumaric and ferulic acids, maintaining cell turgor and an ionic barrier to alleviate stress damage, as reported in maize and melon [86,87].

The principal C6C3C6-compounds that accumulated in *K. scoparia* were naringenin, quercetin, genistein, petunidin, and luteolin (Figure 5). These flavonoids exhibit multidimensional protective effects during plant responses to saline–alkaline stress. C6C3C6-compounds directly scavenge ROS via their –OH groups and activate the antioxidant enzyme network [88,89]; they also chelate Na^+^ to alleviate Na toxicity and maintain potassium channel protein activity, thereby precisely regulating ionic homeostasis under saline–alkaline stress [90]. In *Zea mays*, *ZmWRKY82* enhances saline–alkaline tolerance by promoting *ZmCHI6* transcription and flavonoid synthesis [59], and in alfalfa the MsMYB206–MsMYB450–MsHY5 complex confers salt tolerance through the circadian regulation of flavonoid biosynthesis [91]. Additionally, naringenin, quercetin, genistein, and luteolin have been reported to recruit rhizosphere microbes to combat salt stress [92,93]. Naringenin, quercetin, and luteolin can enhance cell wall mechanical strength and reduce Na transport under saline–alkaline stress [92]. Phenolic compounds can also release H^+^ to neutralize OH^−^, effectively mitigating alkaline stress, especially petunidin and quercetin.

## 5. Conclusions

Plant responses to saline–alkaline stress are complex and multifaceted. In this study, we analyzed the response mechanisms of *K. scoparia* using ionomics and untargeted and targeted metabolomic techniques. The response mechanism differed from that of *S. salsa*; Ca, Mg, and B abundantly accumulated in *K. scoparia* to relieve ion toxicity and oxidative damage, maintain cell stability, and alleviate stress. In addition, Cu and Ni significantly accumulated in the leaves of *K. scoparia*. These elements function synergistically, maintaining the stability of photosynthesis and ensuring normal nitrogen metabolism. Long-term metabolic reprogramming under saline–alkaline stress leads to the accumulation of sugars, phenolic compounds, esters, and alcohols in *K. scoparia* to mitigate salt stress (Figure 6). Further research and analysis revealed that five C6C1-compounds, five C6C3-compounds, and five C6C3C6-compounds significantly accumulated in *K. scoparia* (Figure 6). These compounds enhance ROS scavenging, regulate ion homeostasis and signaling, remodel cell wall architecture, recruit beneficial rhizosphere microbiota and—particularly through flavonoid-mediated H^+^ release—alleviate alkali stress.

## Data Availability

All data generated or analyzed during this study are included in this published article.

## Supplementary Materials

The following supporting information can be downloaded at: https://www.mdpi.com/article/10.3390/plants14223540/s1, Table S1: The values of soil indicators of *S. salsa* community; Table S2: The accumulation of elements in *K. scoparia*, *S. salsa*, and *P. tenuiflora*; Figure S1: Relative content of L-phenylalanine in *K. scoparia*.
