# Supplementary material for: The Ionic and Metabolic Response Mechanisms of Kochia scoparia in Response to Saline–Alkaline Stress
Source: Plants (Basel). 2025 Nov 20;14(22):3540. doi: 10.3390/plants14223540 (PMC12655931; doi:10.3390/plants14223540)
Supplement: Supplementary file 1 [file plants-14-03540-s001.zip › plants-3967381-supplementary.pdf]

Supplementary Materials

Table S1. The values of soil indicators of *S. salsa* community [31].

	pH	ESP (%)	SSC (%)	EC (mS/cm)	CO_3^{2-} (%)	HCO_3^- (%)	CL^- (%)	SO_4^{2-} (%)	Na^+ (%)	K^+ (%)	Ca^{2+} (%)	Mg^{2+} (%)
Ss	9.42	54.87	0.63	1074	0.007	0.010	0.241	0.136	0.010	0.275	0.064	0.186

Ss: the rhizosphere soil around *S. salsa*, ESP: exchangeable sodium percentage, SSC: Soluble salt content, EC: electrical conductivity.

Table S2. The accumulation of element in *K. scoparia*, *S. salsa* and *P. tenuiflora*.

	K	Na	Ca	Mg	Fe	B	Mn	Cu	Zn	Mo	N
KS-L1	22.3375	18.3063	13.5188	12.0563	0.2798	0.0321	0.1059	0.0245	0.0224	0.0026	0.0088
KS-L2	19.9438	15.4438	12.3125	14.1938	0.2631	0.0304	0.1246	0.0270	0.0228	0.0024	0.0065
KS-L3	21.1406	16.8750	13.1750	13.7500	0.2714	0.0349	0.1288	0.0195	0.0226	0.0025	0.0076
KS-S1	14.7125	7.8000	6.3438	3.5063	0.0776	0.0183	0.0809	0.0111	0.0383	0.0013	0.0040
KS-S2	15.4875	11.6813	7.2688	3.2000	0.0794	0.0176	0.0839	0.0114	0.0373	0.0020	0.0046
KS-S3	13.9938	9.7375	7.4813	3.4063	0.0783	0.0188	0.0830	0.0109	0.0378	0.0046	0.0041
KS-R1	6.1688	7.5500	5.9250	6.8063	0.0469	0.0464	0.0676	0.0309	0.0195	0.0045	0.0068
KS-R2	6.3250	9.6063	6.1688	6.1938	0.0461	0.0380	0.0688	0.0311	0.0189	0.0049	0.0069
KS-R3	6.2188	11.7000	6.0438	6.1563	0.0468	0.0456	0.0684	0.0305	0.0190	0.0043	0.0074
PT-L1	9.8438	7.0438	3.9250	2.2313	0.3378	0.0425	0.0901	0.0135	0.0120	0.0040	0.0036
PT-L2	9.8063	6.9313	4.0313	2.2875	0.3451	0.0428	0.0920	0.0135	0.0118	0.0043	0.0039
PT-L3	9.9938	7.4625	3.8750	2.2125	0.3329	0.0421	0.0889	0.0135	0.0119	0.0041	0.0036
PT-S1	8.6500	2.9813	1.7625	0.8813	0.0744	0.0145	0.0674	0.0093	0.0130	0.0026	0.0028
PT-S2	8.6625	2.5438	1.2938	0.7125	0.0659	0.0138	0.0650	0.0091	0.0155	0.0024	0.0028
PT-S3	8.6563	2.7625	1.5281	0.7969	0.0701	0.0141	0.0662	0.0092	0.0143	0.0025	0.0028
PT-R1	4.1188	5.2375	4.1000	3.4625	0.0425	0.0183	0.2058	0.0134	0.0221	0.0031	0.0078
PT-R2	4.1125	5.1563	4.0938	3.5000	0.0425	0.0176	0.2051	0.0135	0.0225	0.0030	0.0074
PT-R3	4.0250	5.2188	4.0250	3.3563	0.0424	0.0188	0.2030	0.0135	0.0216	0.0031	0.0083
SG-L1	32.0125	19.7188	8.7875	10.0000	0.4446	0.0434	0.0893	0.0136	0.0353	0.0043	0.0049
SG-L2	37.2250	19.0750	7.6500	11.5250	0.4293	0.0421	0.0880	0.0146	0.0181	0.0046	0.0046
SG-L3	36.5313	23.1875	11.8063	11.2625	0.4348	0.0443	0.1099	0.0143	0.0318	0.0036	0.0044
SG-S1	22.8438	12.9000	6.4688	6.4438	0.0736	0.0138	0.0833	0.0085	0.0626	0.0030	0.0038
SG-S2	24.6688	12.7875	7.5688	6.1313	0.0890	0.0194	0.0891	0.0090	0.0561	0.0031	0.0044
SG-S3	22.7438	13.0188	6.8438	5.8000	0.0716	0.0369	0.1108	0.0056	0.0556	0.0043	0.0046
SG-R1	11.4375	13.4688	7.0938	10.0000	0.0543	0.0145	0.0880	0.0471	0.0306	0.0030	0.0084
SG-R2	10.4875	12.8250	7.5688	11.5250	0.0598	0.0138	0.0974	0.0480	0.0318	0.0031	0.0081
SG-R3	11.7375	13.5688	6.8438	11.2625	0.0456	0.0141	0.0888	0.0401	0.0335	0.0030	0.0084

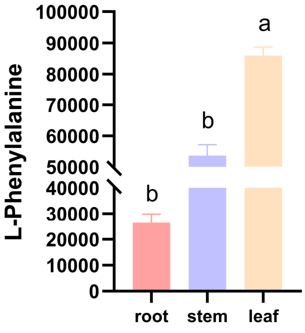

Figure S1. Relative content of L-phenylalanine in *K. scoparia*.
